# Synergistic Effect of Benzethonium Chloride Combined with Endoxan against Hepatocellular Carcinoma in Rats through Targeting Apoptosis Signaling Pathway

**DOI:** 10.31557/APJCP.2020.21.6.1709

**Published:** 2020-06

**Authors:** Omayma A R Abozaid, Fatma S M Moawed, Mostafa A Farrag, Ragaa S M Kawara

**Affiliations:** 1 *Department of Biochemistry, Faculty of Veterinary Medicine, Benha University, Egypt. *; 2 *Health Radiation Research, National Center for Radiation Research and Technology, Atomic Energy Authority, Cairo, Egypt. *; 3 *Radiation Biology, National Center for Radiation Research and Technology, Atomic Energy Authority, Cairo, Egypt. *

**Keywords:** HCC, benzethonium chloride, endoxan, apoptosis, cytchrome C, P53

## Abstract

Combination therapy has been the trendy of care, particularly in cancer remedy, since it is a rational approach to increase response and tolerability and to diminish resistance. Hence, there is a growing interest in combining anticancer drugs to maximizing efficacy with minimum systemic toxicity through the delivery of lower drug doses. Therefore, in the present study, the value of combination between benzethonium chloride (benzo) and endoxan (endo) as anti-tumor drug sensitization of hepatocellular carcinoma HCC treatment were detected both in vitro and in vivo. Crystal violet test was performed to detect the proliferation of HepG2 cells treated with benzo or/and endo. In addition, the HCC rat model was established by diethylnitrosamine (DEN) administration. The antitumor effect was enhanced with the combined treatment of the two drugs, particularly in the group with benzo and endo. The results confirmed that the HCC condition was developed in response to lower expressions of caspase 3 and P53 which, in turn, was due to the overexpression of Bcl-2, and downregulation of cytochrome C. The treatment with benzo combined with endo caused significant activation of caspase-3 mediated apoptotic signals that could be responsible for its anti-HCC potential. Meantime, benzo combined with endo treatments could reduce the hepatocellular carcinogenesis by reducing the expression of MMP-9. Therefore, benzo and endo treatments may be a hopeful therapeutic drug for HCC. Also, more studies are recommended to feat the idea of this research for medical use.

## Introduction

Hepatocellular carcinoma (HCC) is primary cancer represents more than 90% of liver cancers, being a hostile tumor with anticipated cruel survival between 6 and 20 months (Byam et al., 2013). It was reported that HCC is the fifth most frequently diagnosed malignancy and the second major common cause of cancer mortality worldwide (Siegel et al., 2015). Among males, HCC is more predominant than females (Ferlay et al., 2015). In the past 10 years, HCC is believed to be a major health problem with a continuous rise in its incidence rate particularly in Egypt (Abdel Wahab et al., 2017). Chronic hepatitis C virus infection is the major risk factor for HCC with the rising rate in Egypt.

In spite of great progress in antineoplastic drugs, their constrained curative efficacy still remains a clinical obstacle, which is generally ascribed to multi-drug resistance (MDR), induced by conventional drugs and new “targeted” drugs. Other situation also MDR is occurred such as the hypoxic condition inside the solid tumor, making exploring of MDR mode of action is highly required. Several studies have revealed that MDR is associated with overexpression of certain drug efflux pumps, epithelial- mesenchymal transition, the hypoxia-inducible factor1-α signaling pathway, DNA damage repair, autophagy induction and epigenetic regulation (Wen et al., 2016).

**Figure 1:**
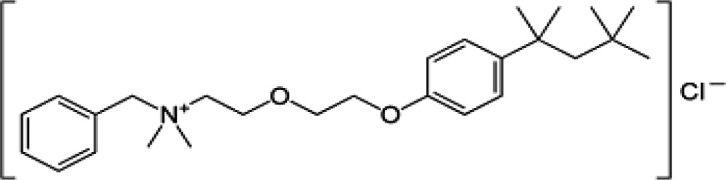
Structure of Benzethonium Chloride

Benzethonium chloride (benzo) is an artificial ammonium salt that is used broadly (Figure 1). Because of their antimicrobial and cationic surfactant properties, benzo is presented widespread in the environment. It has been widely used as preservative agent in vaccines and drugs, phase transfer catalyzers in industry and disinfectants in hospital (Nomura et al., 2010). Also, benzo is present in the baby bath, eye makeup, contact lens, personal hygiene, fragrance, hair, shaving, and skin and suntan preparations as well as in fabric softening, ore flotation, corrosion inhibition and paper processing. Moreover, benzo is found in several grape fruit seed extract preparations and can be used as a preservative (Takeoka et al., 2001), such as in the anesthetic Ketamines (Coates and Flood, 2001). In addition to its environmental uses, benzo was identified as a novel specific anti-cancer agent by using a cell-based small molecule screen (Yip et al., 2006).

Cyclophosphamide or endoxan (endo) is a synthetic anticancer drug that belongs to the nitrogen mustard group of alkylating agents. It acts by attaching an alkyl group to the guanine base of DNA causing irreversible cross-linkages in the DNA strands that lead to cell death or cell-cycle arrest in G2- and S-phases (Poblador et al., 1989). Cyclophosphamide is used extensively in a variety of carcinomas singly or in combination with other drugs to treat a variety of carcinomas due to its cytotoxic property. Cyclophosphamide is a prodrug that gets activated to alkylating phosphoramide mustard in the liver and excreted primarily (70%) in urine in forms of metabolites (Khorwal et al., 2017; Wu et al., 2009).

Hence, the present study was conducted to evaluate the efficiency of the benzethonium chloride combined with endoxan to reduce the incidence of hepatocellular carcinoma. The work comprises both in vitro and in vivo studies. The in vitro cytotoxic effects of this compound were investigated using human hepatocellular carcinoma (HepG-2) cell line. The compounds were further promoted into in vivo studies, using liver-induced tumors in a male animal model, to validate the combination between benzethonium chloride and endoxan could be used as a promising strategy for liver cancer therapy. 

*Chemicals*

Diethylnitrosamine (DEN) and benzethonium chloride (Benzo) were obtained from Sigma Aldrich Chemical Co., St. Louis, Mo. USA. Endoxan (Endo) was obtained from a commercial pharmacy. However, the other chemicals and reagents were used of analytical grade. 

*Animals*

In this study, male Wister rats were obtained from the Nile Pharmaceutical Co., Cairo, Egypt weighing 100–120 g. They were housed at the animal facility at the National Center for Radiation Research and Technology. Before starting the experiment, the animals were allowed to acclimatize for one week and were kept under standard laboratory conditions of light/dark cycle (12/12 h), a temperature of 25 ± 2°C and humidity of 60 ± 5%. The rats were housed in cages with free access to food and drinking water ad libitum. The rats were provided with a standard laboratory (pellet) diet. Meanwhile, the study was conducted in accordance with international guidelines for animal experiments and approved by the Ethical Committee at the National Center for Radiation Research and Technology (NCRRT), Atomic Energy Authority, Cairo, Egypt.

*In vivo study*

Chemical induction of hepatocellular carcinoma:

Animals were orally administered DEN (dissolved in 0.9% normal saline), in a dose of 20 mg/kg b.w. five times a week for eight weeks according to the modified method of Darwish and El-Boghdady (2011). 

*Experimental Design*

Eighty Wister rats weighing 100-120 g were divided into eight groups (10 rats of each one) and classified as follow: 

Group I (Control): Untreated control group.

Group II (Benzo.): Rats received 5 mg/ kg body weight of benzethonium chloride interaperitonealy for 4 weeks (Yip et al., 2006).

Group III (Endo): Rats received 100 mg/ kg body weight of endoxan intraperitoneally for 4 weeks (Wu et al., 2009). 

Group IV (DEN): Rats administered 20 mg/kg b.w. of DEN orally (five times per week for 8 weeks) and left alive for 4 weeks.

Group V (DEN+ Benzo.): Rats administered DEN as in-group IV then injected with benzethonium chloride as in-group II.

Group VI (DEN+ Endo): Rats administered DEN as in-group IV then injected with endoxan as in-group III.

Group VII (DEN+ Benz+ Endo): Rats administered DEN as in-group IV then injected with benzethonium chloride as in-group II and received endoxan as in-group III.

At the end of the experiment, under light anesthesia by diethyl ether, blood samples were withdrawn from the heart of each animal. Then, blood was allowed to coagulate and was centrifuged at 3,000 rpm for 15 min. After blood sampling, animals were sacrificed by cervical dislocation immediately. Part of the liver was promptly removed, washed in ice-cold saline, plotted to dry and weighed for biochemical studies and kept at -80°C until the investigations. The other part of the liver tissue of experimental animals was kept in 10% formalin for histopathological studies.

*Biochemical studies*

*In vitro Cytotoxicity study*

The half -maximal inhibitory concentration (IC50) of benzethonium chloride, endoxan and combination of them were investigated on the viability of the human liver cancer cell line (HepG-2) using cell viability assay by crystal violet staining method.

*Cell line*

Human hepatocellular carcinoma (HepG-2) cell line was used for in vitro investigation of the cytotoxicity of the tested compounds. HepG-2 cell line was obtained from the American Type Culture Collection (ATCC, Rockville, MD cells) and were grown in RPMI-1640 medium supplemented with 10% inactivated fetal calf serum and 50µg/ml gentamycin. The cells were maintained at 37ºC in a humidified atmosphere with 5% CO_2_ and were sub-cultured two to three times a week.

*Cell viability assay*

The antitumor activity was evaluated on carcinoma cell lines at the center for Mycology and Biotechnology, Al-Azhar University, and Cairo, Egypt. In the growth medium, cell lines were grown as monolayers of 10.000 cells. The monolayers are adhered to the bottoms of the wells in a 96-well microtiter plate (falcon, NJ, USA) incubated for 24 h at 37ºC a humidified incubator with 5% CO_2_. Sterile phosphate-buffered saline (0.01 M pH 7.2) was used to wash the monolayers and simultaneously the cells were treated with 100 µl from different dilutions of the tested compound in fresh maintenance medium and incubated at 37ºC. The untreated control cells were made in the absence of the tested compound. For each concentration of the test sample, three wells were used. Every 24 h the observation under the inverted microscope was made. By staining the cells with crystal violet stain, the number of the surviving cells was determined. The cells followed by cell lysing using 33% of glacial acetic acid for the complete cell lysis. The absorbance values were read and the absorbance of untreated cells was considered as 100% proliferation.

*Calculation*

Cytotoxicity (%) = [1 - (OD_T_ / OD_C_)] X 100

Where; OD_T_= mean optical density of tested sample.

OD_C_= optical density of cell control.

*In vivo studies*

*Determination of liver function in serum*

Serum alanine aminotransferase (ALT), aspartate aminotransferase (AST), alkaline phosphatase (ALP), total protein, total bilirubin and albumin were determined using commercially available kits (Spinreact, Santa Coloma, Spain). 

*Determination of Alpha-fetoprotein (AFP) concentration in serum*

Serum alpha-fetoprotein was estimated by enzyme linked immunosorbent assay (ELISA) using a rat alpha- fetoprotein (AFP) ELISA kit purchased from Glory Science Co., Ltd (USA). 

*Determination of caspase -3 in liver tissue homogenate*

Liver caspase -3 was estimated by a rat ELISA kit purchased from Biomatik, Ontario, Canada. 

*Determination of Bcl-2 in liver tissue homogenate*

Liver Bcl-2 was estimated by rat ELISA kit purchased from Biomatik, Ontario, Canada. 

*Determination of P53 gene in liver tissue homogenate*

Liver P53 gene was estimated by rat ELISA kit provided by MyBiosource, San Diego, California, USA. 

*Determination of cytochrome c level in liver tissue*

Liver cytochrome c was estimated by rat ELISA kit provided by MyBiosource, San Diego, California, USA.

*Western blotting of Matrix Metalloproteinase-9 (MMP-9) protein*

Liver tissue proteins were extracted using TRIzol reagent (Invitrogen), and protein concentration was measured according to Bradford (1976). 20 μg of proteins per lane were separated with 10% SDS-PAGE and transferred onto PVDF membranes. In blocking solution (5% non-fat dried milk in 10 mM Tris–Cl, pH 7.5, 100 mM NaCl, and 0.1% Tween 20), the membranes were incubated at room temperature for 2 h and then incubated overnight at 4°C with rabbit IgG MMP-9 antibody (Cell Signaling Technologies, USA) and β-actin antibody (Proteintech, USA) as loading control. After washing three times in 10 mM Tris–Cl, pH 7.5, 100 mM NaCl, and 0.1% Tween 20, the membranes were incubated with HRP-conjugated goat anti-rabbit IgG (Cell Signaling Technologies, USA) at room temperature for 2 h, and then the membranes were washed four times with the washing buffer. According to the manufacturer’s protocols, Amersham detection kit was used to detect the developed membranes and visualized through chemiluminescence. The membranes were exposed to X-ray film. MMP-9 protein was quantified using a scanning laser densitometer analysis (Biomed Instrument Inc., USA).

*Histopathological Study*

Liver tissues from all groups were examined from all groups were examined, washed and dehydrated in ascending concentrations of ethyl alcohol. Then the liver tissues were cleared in xylene and embedded in paraffin wax. Liver tissues were cut out in sections with thickness of 5–6 μm, then deparaffinized and stained with Hematoxylin and Eosin (H and E). Under light microscope stained slides were examined (Banchroft et al., 1996).

*Statistical Analyses*

 Data were analyzed using the SPSS (version 20) with one-way analysis of variance (ANOVA) followed by a post hoc test (LSD) for multiple comparisons. Also, data were expressed as mean ± standard error (SE). P values < 0.05 were considered statistically significant.

**Table 1:** Antitumor Cytotoxic Effects of Different Concentrations of Tested Compounds on Hepatocellular Carcinoma Human Cell Line (HepG-2)

Drug concentration (µg/ml)	Viability % of benzethonium chloride	Drugs concentration (µg/ml)	Viability % of benzethonium chloride combined with endoxan
500	3.48	500	4.01
250	6.31	250	7.83
125	11.94	125	13.45
62.5	23.18	62.5	18.7
31.25	32.65	31.25	25.34
15.6	40.92	15.6	34.57
7.8	48.76	7.8	40.92
3.9	61.39	3.9	46.38
0	100	2	61.79
		1	73.82
		0	100

**Table 2:** Effect of Endo and/or Benzo on Liver Function Test on Rats

	ALT (U/L)	AST (U/L)	ALP (U/L)	Total protein (mg/dl)	Albumin (mg/dl)	Total bilirubin (mg/dl)
Control	14±1.0	9.5±0.5	114.9±1.3	7.1±0.20	5.05±0.15	0.99±0.03
Endo	15±1.0 ^b^	11±1.0^b^	123.9±4.6	7.50±0.30^b^	5.23±0.17^b^	0.95±0.02^b^
Benzo	16±2.0^b^	10±1.0^b^	118.9±2.4	6.50±0.1^ab^	5.45±0.15^b^	0.93±0.02^b^
DEN	67.5±5.5^a^	40.5±1.5^a^	250.5±32.7	4.02±0.01^a^	3.65±0.15^a^	2.6±0.30^a^
Endo+DEN	39.5±1.5^ab^	23±2.0^ab^	178.3±3.1^ab^	6.98±0.08^b^	4.13±0.07^ab^	1.35±0.05^b^
benzo+DEN	40.5±1.5^ab^	24.5±2.5^ab^	163.3±10.4^ab^	7.06±0.04^b^	4.60±0.10^ab^	1.60±0.10^ab^
Endo +benzeo+DEN	27.5±2.5^ab^	19±1.0^ab^	141.5±1.3	7.40±0.1^b^	4.80±0.10^ab^	1.07±0.10^b^

Results are mean ± SE of 8 rats. a and b denote significant change at p<0.05 versus control and DEN groups, respectively. Control, normal control rat; Benzo, Rats received benzethonium chloride interaperitonealy; Endo, Rats received endoxan intraperitoneally; DEN, Rats administered of DEN orally; DEN+ Benzo: Rats administered DEN then injected with benzethonium chloride. DEN+ Endo: Rats administered DEN then injected with endoxan

**Figure 2:**
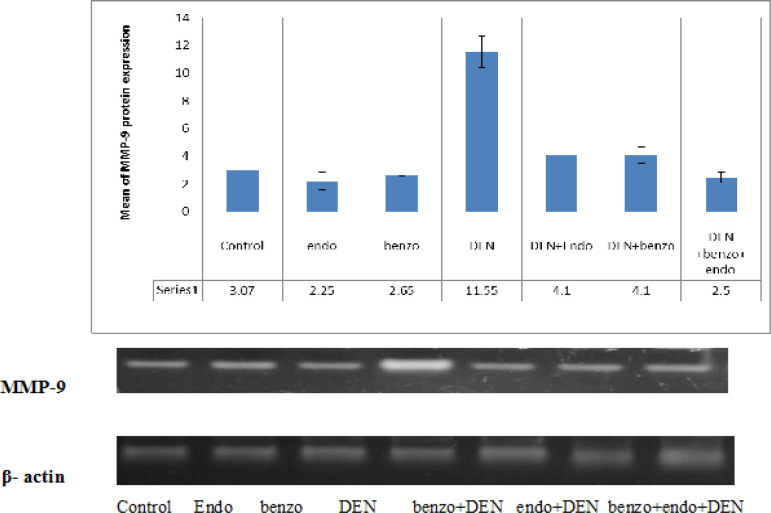
Western Immune Blotting Analysis of MMP9 Protein Expressions in Different Groups

**Table 3:** Effect of Endo and/or Benzo on Tumor Marker and Apoptotic Biomarkers on Rats

	AFP (ng/ml)	Caspase-3 (ng/mg)	Bcl-2 (μg/mg)	P 53 (ng/ml)	Cytochrome C (ng/mg)
Control	0.96±0.0.06	2.36±0.35	20.6±0.75	45.8±3.2	31.4±6.8
Endo	0.85±0.04^b^	2.18±0.12	35.4±8.2^ab^	43.9±3.45^b^	29.8±7.7
Benzo	0.83±0..05^b^	2.25±0.15	27.45±2.35^b^	42.4±1.2^b^	28.1±1.3
DEN	2.55±0.05^a^	1.05a±0.02	76.5±2.7^a^	18.3±4.35^a^	11.65a±1.45
Endo+DEN	1.2±0.10^ab^	5.55±0.75^ab^	35.45±3.15^ab^	42.0±3.75^b^	84.15±8.95^ab^
benzo+DEN	1.15±0.05^ab^	4.7±0.2^ab^	37.55±1.65^ab^	37.7±4.1^b^	82.5±3.9^ab^
Endo +benzeo+DEN	0.96±0.03^ab^	6.9±0.6^ab^	23.35±1.45^b^	44.9±1.3^b^	103.75±6.45^ab^

Results are mean ± SE of 8 rats. a and b denote significant change at p<0.05 versus control and DEN groups, respectively. Control, normal control rat; Benzo, Rats received benzethonium chloride interaperitonealy; Endo, Rats received endoxan intraperitoneally; DEN, Rats administered of DEN orally; DEN+ Benzo, Rats administered DEN then injected with benzethonium chloride; DEN+ Endo, Rats administered DEN then injected with endoxan

**Figure 3:**
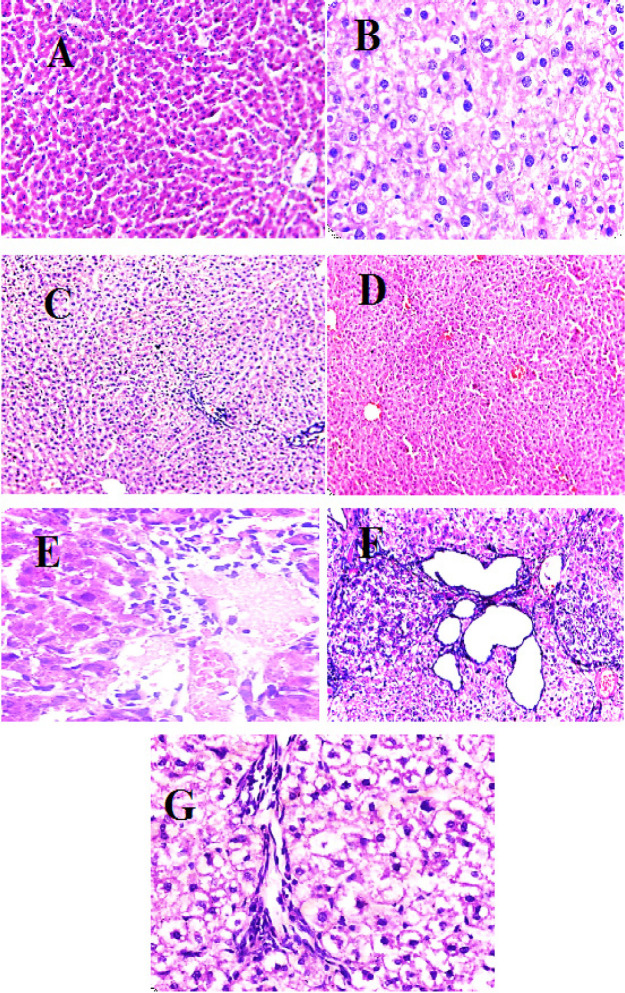
Light Microscopy of Liver Sections Showing: (A) Control group; A normal rat liver tissue), (B) DEN-treated group; A liver of rat receiving DEN showing hepatocellular carcinoma (note the cellular and nuclear pleomorphism, irregular nuclear membranes, coarse chromatin and nuclear inclusions) (H&E x400). (C) Endo-treated group; A liver of rat receiving endoxan showing area of fatty degeneration, dilated congested central veins and periportal inflammatory infiltrate (H&E x100) (D) Benzo-treated group: A liver of rat receiving benzethonium chloride showing dilated congested central vein and sinusoids (H&E x100) (E) DEN+Endo treated group; A liver of rat treated with endoxan after receiving DEN showing hepatocellular carcinoma with dilated vascular channels associated with degenerative changes and chronic inflammatory infiltrate (H&E x200). (F) DEN+ Benzo-treated group; A liver of rat treated with benzethonium chloride after receiving DEN showing hepatocellular carcinoma with degenerative changes, congested portal blood vessels and inflammatory infiltrate (H&E x400). (G) (DEN+Endo+Benzo) An improvement in the tissue liver shape after the synergy between the two treatments ( A synergestic effect was obtained )

## Results

The cytotoxic activity was expressed as the reduction of cell viability relative to control. As shown in Table (1), the combination between benzethonium chloride and endoxan exhibited the strongest in vitro anticancer activity against HepG-2, where, it recorded the lowest IC_50_ value (3.53µg/ml) comparable to that of benzethonium chloride (7.42µg/ml).

*Effects of Endo and benzo on liver function markers*

Data presented in (Table 2) illustrates that rats exposed to DEN displayed a significant increase in serum liver enzymes (ALT, AST and ALP) and total bilirubin, which amounted to 382%, 326.32%, and 118.02% and 162.63% respectively, compared with controls. These increase in hepatic enzymes was accompanied with a significant depletion in total protein and albumin (-43.38% and -27.72%, respectively) compared with control. On the other hand, injection of Endo, benzo either alone or administrated together significantly modulated the alteration in the liver markers compared with DEN group. 

Results are mean ± SE of 8 rats. a and b denote significant change at p<0.05 versus control and DEN groups, respectively. Control: normal control rat, Benzo: Rats received benzethonium chloride interaperitonealy Endo: Rats received endoxan intraperitoneally DEN: Rats administered of DEN orally, DEN+ Benzo: Rats administered DEN then injected with benzethonium chloride. DEN+ Endo: Rats administered DEN then injected with endoxan.

*Effects of Endo and/or benzo on tumor marker and apoptotic biomarkers*

As shown in Table 3, levels of hepatic caspase-3, P53 and cytochrome c with percent change is -55.51%, -56.80% and -62.90% respectively, showed a significant decrease in DEN group compared to the control group. While the levels of AFP and Bcl2 were significantly elevated (165.6%, 271.36%). Treatment with Endo and benzo either alone or together significantly improved the level of AFP and apoptotic biomarkers (Bcl-2, caspase-3, P53 and cytochrome c) compared to DEN treated rats

Results are mean ± SE of 8 rats. a and b denote significant change at p<0.05 versus control and DEN groups, respectively. Control: normal control rat, Benzo: Rats received benzethonium chloride interaperitonealy Endo: Rats received endoxan intraperitoneally DEN: Rats administered of DEN orally, DEN+ Benzo: Rats administered DEN then injected with benzethonium chloride. DEN+ Endo: Rats administered DEN then injected with endoxan

The results in Figure 1 demonstrated that the protein expression of MMP-9 was significantly up-regulated with percent change 276.22% in DEN-exposed rats, as compared to the corresponding normal control ratios (Figure 2). However, the data recognized ameliorations of MMP-9 in the liver of the DEN-treated animals combined with Endo and benzo either alone or together. 

The combination between anticancer drugs is a growing interest that aiming to maximize the efficacy with lower systemic toxicity through the delivery of lower drug doses (Mayer and Janoff, 2007). To our knowledge, the current study is considered the first report showing the anticancer effect of benzo combined with endo against hepatocellular carcinoma. In the current investigation, the results of cytotoxicity revealed that the combination of benzo with endo showed more cytotoxicity against HepG2 cells than benzo when used alone. Furthermore, evaluation of inhibitory percent of cell growth value at 50% (IC_50_=3.53µg/ml) for combination, showed synergism in the inhibition of HepG2 cell proliferation.

Regarding the in vivo study, rats exposed to benzo either alone or in combination with endo reverse the activity of the hepatic enzymes (AST, ALT and ALP), which have been altered by DEN. Since the liver contains an abundance of transaminase groups of enzymes, the activities of both AST and ALT are quite high in hepatocytes. The release of these enzymes in blood takes place from tissues once they get damaged or injured (Saleem et al., 2018). 

Alpha-fetoprotein (AFP) is a potential screening tool that is widely used for the early diagnosis of tumors. It was markedly enhanced in DEN-treated rats compared with normal animals (Chou et al., 2018). In the present study, the obtained results revealed a significant increase in serum AFP levels in DEN-group compared to normal control. Several studies used DEN for HCC induction in albino rats’ revealed similar serum AFP elevation (Metwally et al., 2011; Rajasekaran et al., 2011; Chen et al., 2012). In the current work, injection of benzo and endo attenuated DEN induced hepatocarcinogenesis, as shown by the reverted the activities of liver enzymes and level of AFP when compared to DEN group. This depletion of liver enzymes and the level of AFP might be attributed to the antitumor activity of benzethonium chloride and Endoxan. 

The development and regeneration of liver is regulated by the physiological process called apoptosis (Guicciardi and Gores, 2005). However, its dysregulation is an essential reason for HCC progression (Fabregat, 2009). Apoptosis is managed by numerous signaling pathways along with caspases protease (Yuan et al., 2018). downregulation or loss of caspase-3 expression is in cancerous cell is mostly accompanied by resistance to apoptosis as well as chemotherapy in several kinds of tumors (Kolenko et al., 1999). It was found that the activity of anticancer drugs has been mediated by the induction of apoptosis in target cells (Hickman, 1992: Slee et al., 1999). Also, anticancer drugs activated signaling pathways which ultimately result in activation of caspases (Slee et al., 1999).

In the current study found a significant elevation in caspase-3 activity in benzo and endo treatments alone or in combination groups compared to HCC and control group. These results might be suggested that benzo and endo treatments induce apoptosis. On the other hand, benzo and endo treatments stimulated the intrinsic pathway, caspase-3, was showed from histological result in DEN+ benzo and endo group compared to DEN group.

Yip et al., (2006) reported that benzo might induce the chain of events: First, benzo dysregulates the mitochondria or rough endoplasmic reticulum, which activated caspase within 12 hours. The nucleus begins to condense; rough endoplasmic reticulum swelling continues, and this structure is visibly ‘‘ballooned.’’ The depolarized mitochondria are no longer functional, hence autophagy commences. Finally, at 48 hours, membrane blebbing occurs.

The negative outcomes of cancer therapy could be attributed to the resistance to chemotherapy which is linked to the induction of apoptosis (Hector and Prehn , 2009). Mitochondria has mediated the activation of caspases upon apoptotic stimuli, triggers apoptosis in mammalian cells engage the cell death (Hensley et al., 2013; Hassan et al., 2014). Especially, cytochrome c is released from mitochondria into the cytosol, where it directly activates caspase-3 (Yamamoto et al., 2012). In this study, we observed that benzo and endo treatments alone or in combination enhanced the release of cytochrome c from the mitochondria to the cytoplasm, and increased the expression of cleaved caspase-3 inducing apoptotic cell death.

The obtained results showed that the cellular oxidative damage induced by DEN on rats resulted in a downregulation of p53 and an upregulation pattern for Bcl-2. Reactive oxygen species cause activation and direct mutations in p53, which acts as a tumor suppressor protein (Simabuco et al., 2018). Several types of cancers had lost the function of P53 either gene or protein (Leroy et al., 2013). It plays a pivotal role in removing nuclear and mitochondrial DNA oxidative damage, preventing mutations and genetic instability by mediating DNA repair, cell cycle arrest, senescence and cell death (Danilova et al., 2008). 

Meanwhile, during the survival signal or DNA repair, P53 suppresses the activity of Bcl-2 then extrinsic and/or intrinsic apoptosis initiated (Miyashita et al., 1994). It has been reported that p53 is a potent pro-apoptotic protein, that interacting with Bax protein to induce mitochondrial outer membrane permeabilization (Um, 2016). In the current study, the p53 protein expression was significantly increased accompanied with downregulation of Bcl-2 in benzo and endo treatment groups compared to the DEN-induced HCC group. This indicates that the upregulation of P53 and downregulation of Bcl-2 may have an important role in the regulation of benzo and benzo treatments-induced cell apoptosis. 

The ability of the tumor to invade and migrate regulated the progress of tumor metastasis which is principal characteristics of malignant tumor with poor clinical outcomes. The proteolytic enzymes; matrix metalloproteinase (MMP)-2 and -9 are highly expressed in hepatocellular carcinoma (Helfman et al., 2005), which play important roles in HCC invasion by degrading the environmental extracellular matrix and the basement membrane (Terada et al., 1996).

MMP9 have been established as participants in liver tumor progression. Once the MMP-9 has been disrupted, the mitochondrial permeability will be changed. Also, the pro-apoptotic signals lead to the release of Cyt c from the mitochondria into the cytosol. Cyt c subsequently activates caspase-9, which successively results in the activation of caspase-3 via cleavage induction (Liu et al., 2018). In the present experiment, the levels of MMP-9 were detected in the hepatic tissue of the examined groups. The results showed that benzo+endo treatments significantly reduced the expressions of MMP-9 compared with DEN-treated group, indicating that benzo+endo treatments could suppress the metastasis of liver tumors.

The decrease of MMP-9 after benzo+endo treatments might disrupts the outer mitochondrial membrane, and therefore leads to cytochrome c release from mitochondria and induces apoptosis (Liu et al., 2018). The reduction of MMP-9 causes the nonspecific pores located in the mitochondrial inner membrane to open; the permeability of the matrix then expands and mitochondrial-associated proteins such as cytochrome C (Cyt C) are released (Burlaka et al., 2016). In this work, it was demonstrated that the administration of DEN enhance the up-regulation of MMP-9 while benzo, endo and benzo+endo treatments down-regulate the expression of MMP-9.

In a nutshell, co-administration of benzo and endo has more anticancer activity than using benzo alone against HepG-2 cells. Additionally, benzo might acts synergistically with endo to repress the hepatocarcinogenesis in rat’s model by activating p53 which provoke the apoptosis pathway. Also, benzo and endo treatments could reduce the hepatocarcinogensis by reducing the expression of MMP-9. Nevertheless, further studies are needed to exploit the idea of this research for medical use.
